# Association between parental feeding practices and shared family meals. The Food4toddlers study

**DOI:** 10.29219/fnr.v64.4456

**Published:** 2020-08-05

**Authors:** Nina C. Øverby, Elisabet R. Hillesund, Margrethe Røed, Frøydis N. Vik

**Affiliations:** Department of Nutrition and Public Health, Faculty of Health and Sports Sciences, University of Agder, Kristiansand, Norway

**Keywords:** feeding practices, family breakfast, lunch, dinner, infants

## Abstract

**Background:**

Parental feeding practices and family meals are important determinants for infants’ diet and health. Still, there is no previous research of the association between feeding practices and family meals in infants.

**Objective:**

Explore potential associations between feeding practices and family meals among infants.

**Design:**

We present cross-sectional results (baseline) from the Food4toddlers study. In total 298 parents of 1-year-olds, recruited from all over Norway, filled in a questionnaire regarding frequency of shared family meals (breakfast, lunch, dinner) and feeding practices using the validated instrument Comprehensive Feeding Practices Questionnaire. Logistic regression was used to explore the associations between having family meals every day and feeding practices (10 dimensions).

**Results:**

The children included were about 11 months old, and 55% were boys. Parents were highly educated. Most children had family breakfast and dinner (60–65%), while fewer had family lunch every day (35%). We found that eating family breakfast and lunch every day was associated with use of the positive feeding practices: *encourage balance and variety, environment and modelling* (ORs ranging from 1.15 to 1.37), while eating family breakfast and family lunch less often was associated with the negative feeding practice *pressure to eat* (OR, 95% CI: 0.90 (0.83, 0.96) and 0.91 (0.84, 0.97), respectively). Eating family dinner every day was associated with more use of the positive feeding practice *balance and variety (OR, 95% CI: 1.21 (1.06, 1.38),* while having family dinner less often was associated with use of the negative feeding practices *pressure to eat* and *restriction to health* (OR, 95% CI: 0.89 (0.83, 0.96) and 0.94 (0.87, 1.01), respectively).

**Discussion and conclusion:**

In this group of infants, having family meals every day was associated with positive feeding practices, while having family meals less often was associated with negative feeding practices. Shared family meals provide an important setting for healthy eating, development of feeding skills and dietary habits formation.

## Popular scientific summary

- Family meals and parental feeding practices are important for child diet.
- This study is the first to explore associations between family meals and parental feeding practices in infants.
- We found that having family meals every day was associated with positive feeding practices like *encouraging balance and variety*. Having family meals seldom was associated with negative feeding practices like *pressure to eat*.
- Promoting *both* family meals and positive feeding practices should be prioritized from early age.

A healthy diet during infancy and toddlerhood is essential for healthy growth and development and fundamental for lifelong health (1, 2). Parents are gatekeepers of their child’s diet, both in relation to **what** is served, in **what settings** it is served and **how** it is served (3, 4). Regarding what is served, a diet high in fruits and vegetables is highly recommended together with whole grains, milk and fish (5), however, national data show that it is challenging for parents to include vegetables and fish in their children’s diet (6).

Regarding in what settings food is served, there is an increasing attention related to family meals, however, the focus has primarily been towards older children (7). Family meals are by Verhage et al. defined as eating together as a family (7). There is research showing that family meals are particularly important in relation to a healthy diet and optimal weight development (8). Verhage et al. identified four studies focusing on family meals in infants and toddlers and found that toddlers regularly exposed to family meals had higher odds of eating fruits and vegetables and enjoying meals (7). However, no studies have to our knowledge explored the relation between family meals and parental feeding practices in infants, only in adolescence (7, 9).

Regarding **how** food is served, parental feeding practices are important determinants of child diet. Peters and co-workers define parental feeding practices as parental influence on the development of children’s attitudes and preferences around food and eating (10, 11). It is generally argued that positive feeding practices such as authoritative practices with high scoring on modelling, involvement and encouraging balance and variety are related to a higher child intake of fruits and vegetables and generally a healthier diet (12). While feeding practices like restriction and using food as a reward are associated with reduced self-regulation of child food intake and higher intakes of unhealthy foods in young children (10). In a cohort of preschoolers aged 5–6 years, unhealthy feeding practices including food as a reward for good behaviour and food restriction for promoting health were associated with increased consumption of junk food, sweets, and snacks. In the same cohort, healthy feeding practices like encouraging balance and food variety and modelling were associated with increased vegetable consumption (12). Mixed results are shown regarding controlling feeding practices and child diet (10). Data is, however, scarce and conflicting regarding all these associations among infants and toddlers (13).

With family meals being an important setting for child diet and dietary behaviour, identifying associations between feeding practices and regularity of family meals is important. In this paper, we explore potential association between feeding practices and family meals in families of infants. We used baseline data from the intervention study Food4toddlers (14) to explore these relations.

## Data, methods and subjects

### Study design and recruitment

Food4toddlers is a randomized controlled trial, and we present baseline results in this paper. Parents of infants were recruited to the study with the main aim of investigating the effect of a digital dietary intervention. Parents were recruited through social media (Facebook) posts targeting parents of infants aged 12 months. Parents voluntarily notified that they wanted to participate after reading written information about the study. The study was approved by the Norwegian Centre for research Data, and by the Faculty ethics committee and has been conducted in line with the Helsinki Declaration of 1985, revised 2008 (15). A total of 404 parents agreed to participate initially, however, when the questionnaire was sent to the parents around child age 12 months, 298 parents filled in the baseline questionnaire. These are the ones that contributed data for this paper.

### Methods

Parents filled in a questionnaire with questions regarding background information (gender, relation to child, age, educational level), food frequency questions of child diet, food frequency questions of parental diet, feeding practices measured by Comprehensive Feeding Practices Questionnaire (CFPQ), meal and family meal frequencies and other intervention specific questions. For this paper, background questions and questions regarding family meals frequency and feeding practices are presented. Questions posed regarding family meals were: How often does your child have the following meals together with their family? Response alternatives were given in times per week (never, 1, 2, 3, 4, 5, 6 and 7 times per week) for breakfast, lunch, dinner and evening meal. Weekly frequency was later dichotomized into having each respective family meal every day or not. The evening meal was not included in further analyses since this meal is mostly served outside a family meal setting.

Feeding practices were assessed using Musher-Eizenman et al.’s CFPQ (16). The original version included 44 items and assumed to cover 12 dimensions of parental feeding practices. The CFPQ was originally developed to measure multiple feeding practices used by parents of children in the age span from about 2 to 8 years. In the present study the questionnaire was slightly modified to fit parents of infants. Five items were considered irrelevant to parents of infants and were therefore removed: 1) I involve my child in planning family meals; 2) I encourage my child to participate in grocery shopping (Both belonging to the dimension *Involvement*); 3) I encourage my child to eat less so he/she won’t get fat; 4) I often put my child on a diet to control his/her weight(Both belonging to the dimension *Restriction for weight control*); And 5) I discuss with my child the nutritional value of foods (belonging to the dimension *Teaching about nutrition*)). This led us to not present the dimensions *Involvement* and *Teaching about nutrition* in this paper. We therefore present 10 dimensions of child feeding practices, coded according to Musher-Eizenman and Holub (16). The 10 dimensions are defined as follows: *Child control* (Parents allow the child control of his/her eating behaviours and parent–child feeding interactions), *Emotion regulation* (Parents use food to regulate the child’s emotional states), *Encourage balance and variety (*Parents promote well-balanced food intake, including the consumption of varied foods and healthy food choices), *Environment* (Parents make healthy foods available in the home), *Food as reward* (Parents use food as a reward for child behaviour), *Modeling* (Parents actively demonstrate healthy eating for the child), *Monitoring* (Parents keep track of child’s intake of less healthy foods), *Pressure* (Parents pressure the child to consume more food at meals), *Restriction for health* (Parents control the child’s food intake with the purpose of limiting less healthy foods and sweets) and *Restriction for weight control* (Parents control the child’s food intake with the purpose of decreasing or maintaining the child’s weight) (16). Details of the reliability, example of questions asked for each dimension and mean score are given in Table 1. The CFPQ items were previously translated from English into Norwegian in another project and a random sample of 10 items were back-translated into English (17). The quality of the translation was considered very good as the meaning of the items were retained after translation/back translation and this translation was therefor used in this project.

**Table 1:** The 10 dimensions included from the Child Feeding Practices Questionnaire with mean (SD) score, reliability assessment (α), and number of items with examplec

Dimension	Mean (SD)	α	Number of items and example
Child control	6.4 (2.8)	0.34	5 items (*Do you let your child eat whatever s/he wants?*)
Emotion regulation	3.3 (1.8)	0.63	3 items (*When this child gets fussy, is giving him/her something to eat or drink the first thing you do?*)
Encourage balance and variety	14.3 (1.8)	0.47	4 items (*I encourage my child to try new foods*)
Home environment	12.3 (3.1)	0.68	4 items (*Most of the food I keep in the house is healthy*)
Food as a reward	1.2 (1.6)	0.33	3 items (*I offer sweets to my child as reward for good behaviour*)
Modelling	13.2 (2.8)	0.67	4 items (*I model healthy eating for my child by eating healthy foods myself*)
Monitoring	14.6 (2.8)	0.80	4 items (*How much do you keep track of the high-fat food that your child eats?*)
Pressure to eat	6.4 (3.5)	0.67	4 items (*My child always eats all of the food on his/her plate*)
Restriction for health	5.7 (3.2)	0.51	4 items (*If I did not guide or regulate my child’s eating, he/she would eat too many junk foods*)
Restriction for weight control	6.2 (4.2)	0.64	6 items (*I restrict the food my child eats that might make him/her fat*.)

The parents reported their own age, child age and gender. They also reported level of education for themselves and the other parent (primary school or less, primary schools plus 1 year of further education, high school, vocational school, upper secondary school or less, college/university (≤4 years), college/university (>4 years), other, don’t know). These responses were dichotomized into having no university/college education or having university/college education.

### Statistics

Descriptive data are presented with means and SDs and percentages in Table 2. The reliability of the 10 feeding practices dimensions was evaluated using Cronbach alpha. All scores except *Child control* and *Food as a reward* (α = 0.3) showed acceptable reliability (α = 0.5–0.8). To explore the relation between the respective feeding practices and family meals, logistic regression analyses were used. Family meals (breakfast, lunch and dinner, respectively, daily vs. less than daily) were used as dependent variables and all 10 dimensions of feeding practices (continuous scores) were analysed separately in crude models and adjusted models. We adjusted for child age, child gender, parental education (both parents’ education level) and age of the parent filling out the questionnaire in all adjusted models. Adjustment was done according to known covariates of feeding practices and meal frequencies. Assumptions for logistic regression were met. Data is analysed using the statistical package IBM SPSS 25.0. For all tests, *P* ≤ 0.05 was considered significant.

**Table 2:** Participant characteristics (mean (SD), or n (%))

Characteristics	Mean (SD) or *n* (%)
**Child characteristics**	
Gender (% girls)	134 (45)
Age, months (mean)	10.9 (1.2)
Breakfast with family^1^	179 (60.1)
Lunch with family^1^	106 (35.6)
Dinner with family^1^	196 (65.8)
Attending kindergarten or day care by grandparents (%)	79 (26.5)
**Parental characteristics**	
Age mother	32.8 (4.2)
Age father	32.3 (4.2)
Maternal education^2^ (% high)	261 (87.6)
Paternal education^2^ (% high)	190 (63.8)

1 Family meals 7 days a week.

2 Education high: University/college education.

## Results

Participant characteristics are given in Table 2. The children included in the study were about 11 (SD 1.2) months old, and 55% were boys. Parental age was 32 years and the majority of both mothers and fathers were highly educated (64 and 88%, respectively). Most children had family breakfast and family dinner (60 and 66%, respectively), while fewer had family lunch every day (36%).

Using the positive feeding practice *encouraging balance and variety* was associated with having breakfast, lunch and dinner every day. Further the positive feeding practices healthy home *environment and modelling* were associated with both having breakfast daily and having lunch daily*.* While use of the negative feeding practice *pressure to eat* was associated with having breakfast, lunch and dinner less often and *restriction for health* was associated with having dinner less often (Table 3).

**Table 3:** Adjusted^1^ associations between increasing score of feeding practices dimensions and family meals (breakfast, lunch and dinner)

Feeding practices	Breakfast		Lunch		Dinner	
	OR (95% CI)	*P*	OR (95% CI)	*P*	OR (95% CI)	*P*
Child control	1.08 (0.99, 1.18)	0.067	0.99 (0.92, 1.08)	0.912	1.07 (0.98, 1.16)	0.151
Emotional regulation	0.94 (0.82, 1.08)	0.355	0.95 (0.83, 1.09)	0.482	1.00 (0.87, 1.16)	0.944
Balance & variety	1.37 (1.18, 1.58)	**<0.001**	1.35 (1.15, 1.59)	**<0.001**	1.21 (1.06, 1.38)	**0.005**
Environment	1.21 (1.11, 1.32)	**<0.001**	1.15 (1.05, 1.25)	**0.002**	1.07 (0.99, 1.16)	0.092
Food as reward	0.95 (0.82, 1.10)	0.486	0.93 (0.80, 1.09)	0.381	0.94 (0.80, 1.09)	0.379
Modelling	1.19 (1.09, 1.31)	**<0.001**	1.16 (1.05, 1.28)	**0.003**	1.06 (0.97, 1.16)	0.196
Monitoring	1.03 (0.95, 1.12)	0.516	1.04 (0.95, 1.14)	0.433	0.94 (0.86, 1.04)	0.241
Pressure	0.90 (0.83, 0.96)	**0.003**	0.91 (0.84, 0.97)	**0.007**	0.89 (0.83, 0.96)	**0.003**
Restriction health	0.99 (0.93, 1.05)	0.989	0.95 (0.88, 1.03)	0.192	0.94 (0.87, 1.01)	**0.017**
Restriction weight	1.00 (0.95, 1.07)	0.857	1.02 (0.96, 1.08)	0.523	0.97 (0.90, 1.03)	0.251

1 Adjusted for maternal and paternal education, age of child, age of the adult filling out the questionnaire.

Significant *P*-values **in bold.** Name of feeding practice dimensions are shortened for table use but described elsewhere in the manuscript.

Since some children attended kindergarten (26%) and therefore were less likely to attend family lunch every day, we performed a sensitivity analysis confined to those being at home with their parents during daytime (74%). We found that the estimates were all in the same direction as presented in Table 3, while two associations differed regarding significance level. The association between *balance and variety* and family dinner was not significant in the subgroup of children not attending day care (OR: 1.15, 95% CI: 0.98, 1.34, *P* = 0.088), while the association between *Restriction for weight control* and family dinner was significant (OR: 0.93, 95% CI: 0.86, 0.99), *P* = 0.045). Further, as our cut off for having family meals every day is quite strict, we reran all regression models using *having 6 family meals a week or more* as the cut off of having family meals often, as a sensitivity analysis. When using this cut off all associations reported for family breakfast and lunch remained the same as the strict definition. However, the associations between having family *dinner and balance* and variety and *restriction for health* were no longer significant, although ORs were in the same directions as before (OR: 1.10 [0.966, 1.26]], *P* = 0.149 and OR:0.93 [0.87, 1.02], *P* = 0.116, respectively). In addition, we found a new association between having dinner often and using the positive feeding practice Involvement (OR: 1.29 [1.07, 1.58], *P* = 0.008).

## Discussion

Our results indicate that in families of infants, family meals were more common when positive feeding practices (balance and variety, home environment and modelling) were used. Further, family meals were less common in families were negative feeding practices like *pressure to eat* and *restriction for weight* were used. This is one of the first studies identifying these associations in young children.

Associations between parental feeding practices and frequency of family meals have previously been described in adolescent households (9, 18), however not among toddlers and infants. We found that those with higher scores on positive feeding practices such as *encouraging balance and variety, home environment* and *modelling*, had higher odds of having family breakfast and lunches. This is overall in line with what has previously been reported in adolescents (9, 18), and although not comparable due to age, this shows that specific feeding practises are related to regularity of family meals at different age points. Our results indicate that these associations are established early in toddlerhood. As for mechanisms, it seems rational that parents who create a healthy home environment and focus on well balanced food intake, also are motivated to having family meals. Family meals may also be their way of fulfilling intentions of a healthy food intake and a beneficial food environment for their children. Modelling has not, to our knowledge, previously been reported to be associated with family meals in adolescents. The observed relationship among infants may easily be explained by a larger opportunity to model eating behaviour while eating together with young children than adolescents. Berge et al. argue that mechanisms behind the associations between positive feeding practices and regularity of family meals may be that a home environment with structure and warmth may promote the occurrence and perceived success of family meals (18). They also argue that occurrence of family meals is a marker of positive feeding practices (18). Frequent family meals may also be an indicator of a health promoting lifestyle in general.

We further found that negative feeding practices such as *pressure to eat* and *restriction for health* were related to lower odds of regular family meals (all three meals and dinner only, respectively). Among adolescents the feeding practice *pressure to eat* was associated with higher frequency of family meals (9). The authors speculated that with higher frequency of meals together, such practice might be easier to uphold. However, one could also argue, that pressure to eat could create a more negative meal atmosphere and therefore not stimulate family meals, and that this feeding practice would be easier to uphold in a one-to-one feeding situation for infants. In the review of Verhage et al. it is reported that mothers mention different mealtime stressors, like the child’s behavior (picky eater or stubborn), as reasons not to maintain family meals (7). In our study, one could speculate that pressure to eat is a parental reaction to picky eating or food neophobia in the child, and that this might be a reason for reduced odds of family meals. There is no obvious reason for why the feeding practice *restriction for health* should be associated with reduced odds of family dinner, as having family meals are known to be healthy. One could speculate that restrictions are part of an authoritarian feeding style that is simultaneously associated with less frequent family meals (19). However, when changing the cut off for having family meals to six meals a week or more, this association was no longer significant.

There has been an increased focus on family meals the last decade, mostly focusing on older children. Verhage et al. recently published a review on data from infants and toddlers showing, although with fewer studies included, that family meals are related to better nutrition, healthier food intake and fewer eating problems (7). This shows the importance of even in toddlerhood prioritizing family meals with both children and parents present. Our study shows that more than 60% have family breakfast and dinner every day in infant families. The lack of previous focus on family meals for the youngest children may possibly be explained by infants being in the transition phase from baby food to family food and that being a part of the family meal setting is new for the infant and toddler. Continued research on what is associated with family meals is important. Our study shows that parental feeding practices and family meals are associated, and that the same pattern of typically unhealthy feeding practices is associated with lower odds of family meals while healthy feeding practices are associated with higher odds of family meals. Norwegian health authorities inform about the importance of family meals in their information for parents of infants and toddlers (20). Our results in addition to what has been reported by Verhage et al., yield further evidence to highlighting this more in public health messaging.

### Strengths and limitations

Strengths of our study is the detailed information about feeding practices, using the validated and well-studied CFPQ, with acceptable reliability. Further we have included relevant background characteristics like parental age and socio-economic background in our analysis. It is further a strength that the included children have a narrow age span, indicating homogeneity in this group where there is lack of information internationally.

There are also limitations to our study. These include the cross-sectional design of the current analysis, which does not allow causal inference. Further, the data are self-reported, which means that there is a possibility of misreporting, however, these measures are best identified using self-reporting, because observation would not be doable in such large numbers of participants. CFPQ is originally made for children aged 2–8 years (16), and therefore our sample is somewhat young. Care was taken to remove questions that were not relevant for the age group 12 months. This means that we excluded five different items resulting in the removal of two dimensions (teaching about nutrition and involvement). Further, one could say that the timing of this study in relation to child age is difficult and may be regarded as a limitation. In Norway, most children start attending kindergarten at the age between 12 and 18 months, some start even earlier, which means that family meals are less likely to occur during daytime (21). To control for this, we performed a sensitivity analysis confined to those not attending day-care and included this in the paper. In addition, the generalizability of our findings is limited because more than 80% were highly educated, being skewed compared to the general population (22). Further, the question regarding family meal, was framed in general, ‘how often does the child eat together with their family’, without stating what that means regarding number of family members attending the meal. However, this is in line of the review of Verhage et al. a family for infants and toddlers are most relevant to be described as *a social moment* of the day during which *food is eaten together with at least one family member* (7).

## Conclusion

Family meals are related to several benefits as improved diet quality, also among infants. In our study, Food4Toddlers among 1 year-old children, we found that having family meals daily was associated with positive parental feeding practices (balance and variety, home environment and modelling), and the opposite, having family meals less often, was associated use of negative feeding practices like pressure and restriction for weight. This is one of the first studies identifying these associations in young children and more research is needed to obtain a better understanding of the mechanisms in play. Understanding predictors for family meals is highly important due to the importance of family meals as a natural setting for promoting a healthy and balanced diet and healthy eating behaviours. Promoting both positive feeding practices and family meals should be prioritized from an early age.

## Data Availability

The dataset supporting the conclusions of this article will be available in the UiA Open Research repository (https://dataverse.no/dataverse/uia).
